# Mapping the intersection of HIV and Alzheimer’s disease: a bibliometric analysis of emerging research trends

**DOI:** 10.3389/fneur.2025.1568022

**Published:** 2025-04-29

**Authors:** Hao Zhang, ShuYou Yuan, HongXia Bao, WenJun Chen, Bo Cai, JunKai Sun, HaoGang Zhu, Wei Lu

**Affiliations:** ^1^Geriatrics Center, Wuxi Second Geriatric Hospital, Wuxi, China; ^2^Laboratory Department, Wuxi Second Geriatric Hospital, Wuxi, China; ^3^Neurology Department, Wuxi Second Geriatric Hospital, Wuxi, China; ^4^Pathology Department, Wuxi Second Geriatric Hospital, Wuxi, China; ^5^Department of Interventional Radiology, Wuxi No. 5 People’s Hospital, Wuxi, China

**Keywords:** HIV, Alzheimer’s disease, bibliometrics, neuroinflammation, protein aggregation, blood–brain barrier, HAND, aging HIV population

## Abstract

**Background:**

HIV and Alzheimer’s disease (AD) are significant global health challenges with overlapping neuroinflammatory and protein aggregation mechanisms. Understanding their intersection is critical for advancing therapeutic strategies, particularly in aging populations.

**Objective:**

This study aims to provide a comprehensive bibliometric analysis of research trends at the intersection of HIV and AD, identify emerging themes, and highlight key contributors in this interdisciplinary field.

**Methods:**

Using the Web of Science Core Collection, we retrieved 4,856 articles and reviews published between 1994 and 2025. Bibliometric analysis was conducted with VOSviewer, CiteSpace, and R software to examine publication trends, international collaboration, institutional contributions, journal dynamics, author networks, and thematic evolution.

**Results:**

The analysis reveals a 14.18% annual growth rate in publications, with the U.S. leading in productivity, followed by China, Germany, and Japan. Key institutions include the NIH and the University of California System, while journals such as *Journal of Biological Chemistry* and *PLOS ONE* show significant growth. Prominent authors include Masliah, Eliezer, and Heaton, RK. Research highlights the overlap between HIV-associated neurocognitive disorders (HAND) and AD, emphasizing shared mechanisms like neuroinflammation, protein aggregation, and blood–brain barrier disruption. Recent advances focus on cerebrospinal fluid biomarkers, oxidative stress, and the impact of antiretroviral therapy (ART) on neurological outcomes. Studies increasingly explore the role of advanced methodologies, including machine learning, in elucidating shared mechanisms such as neuroinflammation, endoplasmic reticulum stress, and protein misfolding.

**Conclusion:**

This bibliometric analysis underscores the dynamic and rapidly evolving research landscape at the intersection of HIV and AD, driven by collaborative efforts and technological advancements. Future research should prioritize longitudinal studies, mechanistic insights, and translational applications to address unanswered questions in this critical field.

## 1 Introduction

This study focuses on the intersection of HIV and AD, two significant global health challenges that collectively affect millions of individuals worldwide (1). HIV, a viral infection that compromises the immune system, has transitioned from a fatal disease to a chronic condition due to advancements in ART (2). However, ART does not fully mitigate the neurological consequences of HIV infection, with HAND remaining prevalent among aging HIV-positive populations (3). AD, a progressive neurodegenerative disorder characterized by cognitive decline, memory loss, and behavioral changes, is the most common form of dementia in older adults (4). Both diseases impose substantial burdens on patients, families, and healthcare systems, contributing to increased morbidity, mortality, and economic costs associated with caregiving and treatment (5). Current diagnostic and therapeutic approaches for these diseases remain limited (6). While ART has transformed HIV management, it does not fully address the cognitive impairments associated with HIV (7). Similarly, AD research has faced challenges in developing effective treatments, with most therapeutic interventions focusing on symptom management rather than disease modification (8). The overlap in neuroinflammation, protein aggregation, and blood–brain barrier disruption between HIV and AD suggests shared pathological mechanisms that warrant further investigation (6). Therefore, this research is necessary to better understand the complexities and interactions between these diseases, particularly in the context of an aging global population and the growing prevalence of comorbid conditions.

Bibliometrics is a quantitative research method that employs mathematical and statistical techniques to analyze the distribution, structure, and trends of scientific literature (9). By examining publication output, citation patterns, author collaborations, and keyword co-occurrence, this method helps researchers understand the development trajectory of a field (10). Bibliometric analysis is particularly useful for identifying research hotspots, emerging trends, and key contributors in interdisciplinary domains such as the intersection of HIV and AD (11). By mapping the intellectual landscape of the field, this method helps researchers prioritize future studies and allocate resources effectively (12). It also aids in understanding how interdisciplinary collaboration and technological advancements shape the research agenda (13). In the context of HIV and AD, bibliometric analysis can identify shared research priorities, such as neuroinflammation, protein aggregation, and blood–brain barrier disruption (14).

This research employs bibliometric analysis methods to systematically review and analyze the literature at the intersection of HIV and AD. Using the Web of Science Core Collection, we retrieved 4,856 articles and reviews published between 1994 and 2025. The advantages of this approach include the ability to visualize trends, collaborations, and key contributions within the scientific community. The objective of this study is to provide a comprehensive overview of the research landscape, identify emerging trends, and highlight the contributions of various authors, institutions, and journals in this field.

## 2 Materials and methods

### 2.1 Data sources and retrieval strategy for HIV and AD bibliometrics

To conduct a comprehensive bibliometric analysis of the intersection between HIV and AD, we utilized the Web of Science Core Collection, a high-quality database for bibliometric research. Our search strategy included English-language research articles and reviews published between 1994 and 2025. The search query was carefully formulated to capture relevant literature: TS = [(“HIV” OR “HIV-1” OR “HIV-2” OR “human immunodeficiency virus” OR “HIV encephalitis” OR “HAND” OR “HIV-associated neurocognitive disorder” OR “neuroAIDS”) AND (Alzheimer* OR “Alzheimer’s disease” OR “Alzheimer’s” OR “Alzheimer disease” OR “Alzheimer’s-like” OR “Alzheimer pathology” OR “Alzheimer pathogenesis” OR “tau protein” OR “beta-amyloid”)]. This search yielded a total of 4,856 documents, which were further filtered to include only research articles and reviews. The complete list of these 4,856 documents, along with the detailed search results, is provided in Supplementary Table 1.

### 2.2 Analytical methods and tools for bibliometric analysis

**Figure 1:**
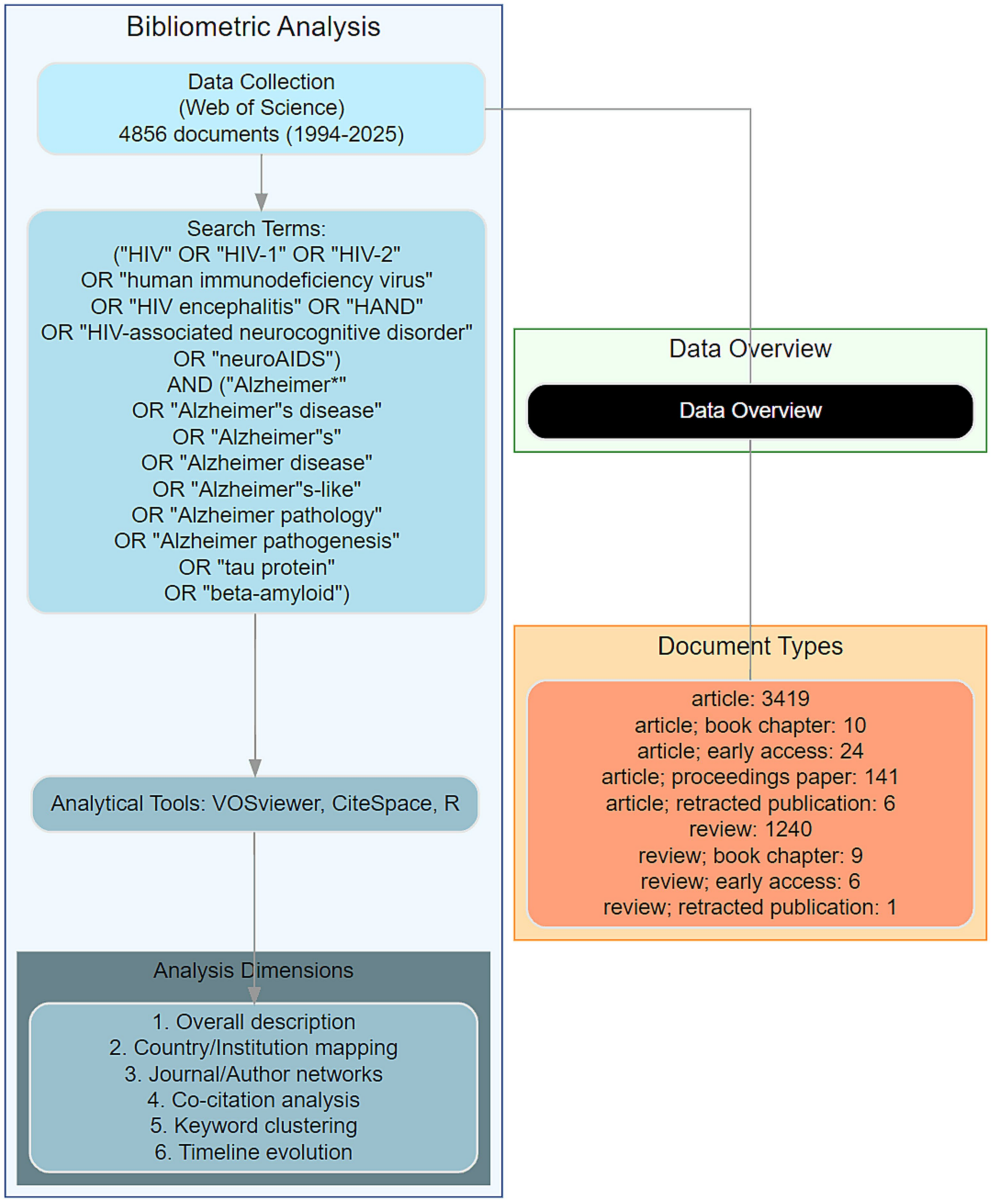
Workflow of bibliometric analysis. A comprehensive schematic illustrating the structured workflow for bibliometric analysis of HIV-Alzheimer’s disease (AD) intersections. The workflow encompasses data retrieval from the Web of Science Core Collection, analytical tool integration (VOSviewer, CiteSpace, and R software), and systematic examination of eight dimensions: overall trends, country-level contributions, institutional collaborations, journal dynamics, author networks, document impact, thematic evolution, and keyword co-occurrence. This workflow underscores the methodological rigor and interdisciplinary approach employed in this study.

To conduct a thorough bibliometric analysis, we employed a combination of advanced tools: VOSviewer (version 1.6.20), CiteSpace (version 6.4 R1), and R software (version 4.3.3). VOSviewer was utilized to create visual representations of bibliometric networks, focusing on the depiction of collaboration between authors, institutions, and countries. It was also used to generate cluster maps of co-cited authors, citations, and keywords, as well as their timeline maps (15). CiteSpace was employed to detect and graphically represent emerging trends and patterns within the scientific literature. It was particularly useful for keyword burst detection and timeline analysis, helping to identify the dynamic evolution of research themes over time (16). R software was utilized to analyze publication data, author productivity changes, journal issues, and institutional contributions. It was particularly useful for plotting annual publications, citations, and citation fits to relevant literature, providing insights into the development trajectories of the field. The analysis process included overall description, country, institution, journal, author, document, topic, and keywords analysis focusing on genes related to HIV and AD. This structured approach ensured a comprehensive and systematic examination of the literature, facilitating a deeper understanding of the research landscape in this area. Our complete analysis workflow is illustrated in Figure 1.

## 3 Results

### 3.1 Overall description of the research landscape

**Figure 2:**
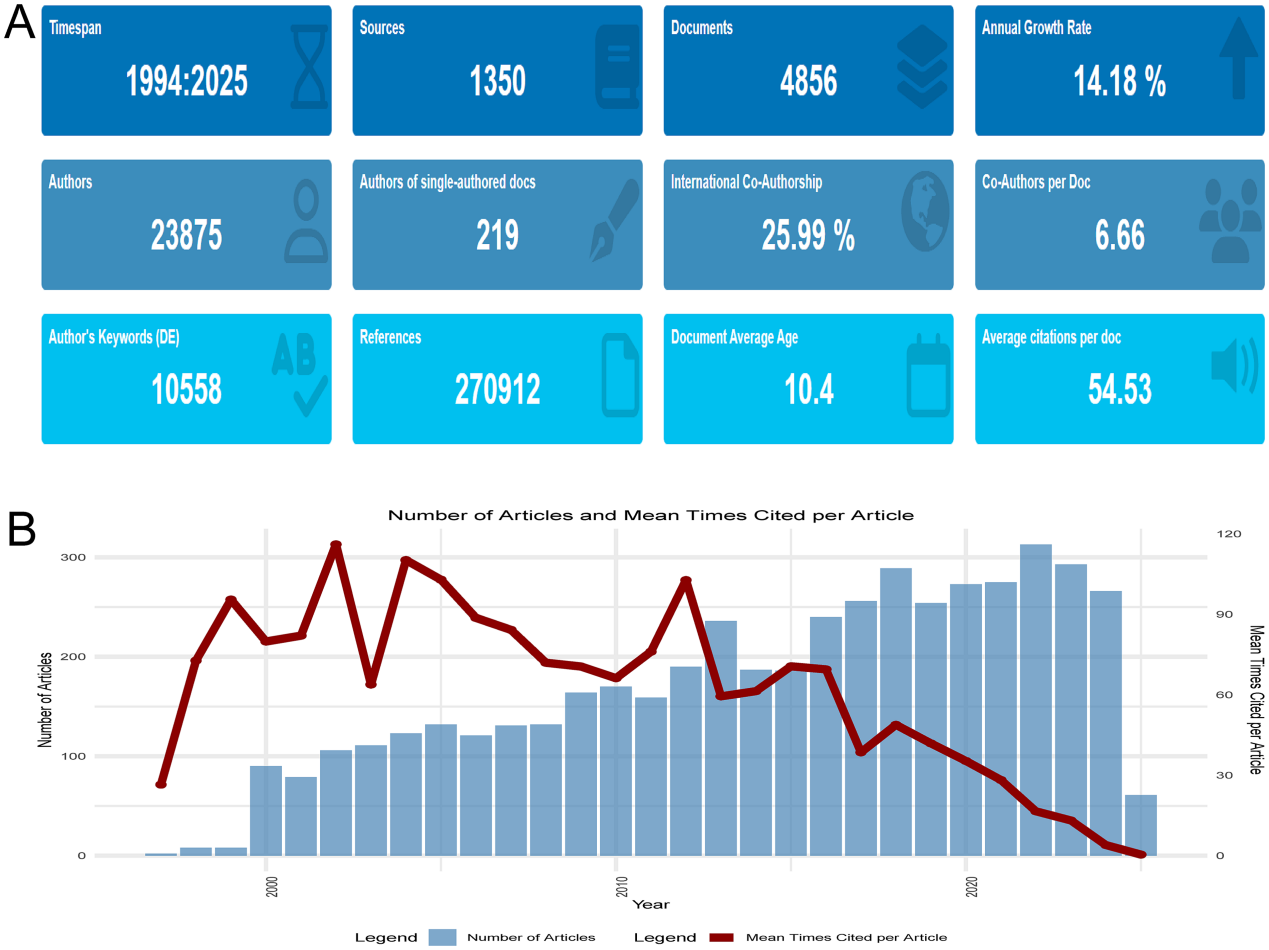
Overall research landscape. **(A)** Temporal trends in publication output (1994–2025), highlighting a 14.18% annual growth rate, collaborative dynamics (25.99% international co-authorship), and average citations per document (54.53). **(B)** Annual publication counts and mean citations per article (1997–2025), illustrating fluctuations in citation metrics. Notable peaks in 2002 (mean citations = 116.08) and declines in recent years (2024 = 3.96, 2025 = 0.41) are attributed to citation lag effects, underscoring the dynamic evolution of research impact over time.

Our study presents a thorough bibliometric analysis of 4,856 research articles and reviews on the intersection of HIV and AD, published from 1994 to 2025 and retrieved from the Web of Science Core Collection. As depicted in Figure 2A, the annual publication output has shown a notable upward trend. Despite the fluctuating annual growth rate of 14.18%, the number of publications has consistently increased over the years, indicating a burgeoning research interest and activity in this field. With 25.99% of the publications being internationally co-authored and an average of 6.66 co-authors per document, this field is highly collaborative, underscoring the global scope of the research. The average citation count per document is 54.53, reflecting the substantial influence and impact of the research in this area. The data in Figure 2A, which includes details on the number of authors, references, and document average age, collectively underscore the dynamic nature and key characteristics of research at the intersection of HIV and AD.

Figure 2B presents a comprehensive overview of the annual publication output and the mean times cited per article from 1997 to 2025 in the field of HIV and AD research. The field witnessed a modest beginning in 1997 with only two articles, each receiving 26.5 citations on average. Over the years, the number of publications has shown a general upward trajectory, reaching a peak of 313 articles in 2022. However, the mean times cited per article has experienced fluctuations. Notably, in 2002, the mean times cited per article reached a high of 116.08, indicating the significant influence of certain seminal studies published that year. In contrast, recent years have seen a decline in the mean times cited per article, dropping to 3.96 in 2024 and 0.41 in 2025. This could be attributed to the recency of these publications and the inherent time lag in citation accumulation. Despite the variations in citation metrics, the overall increasing trend in the number of publications reflects a growing research interest and academic attention in the intersection of HIV and AD.

### 3.2 Country-level analysis of research contributions

**Figure 3:**
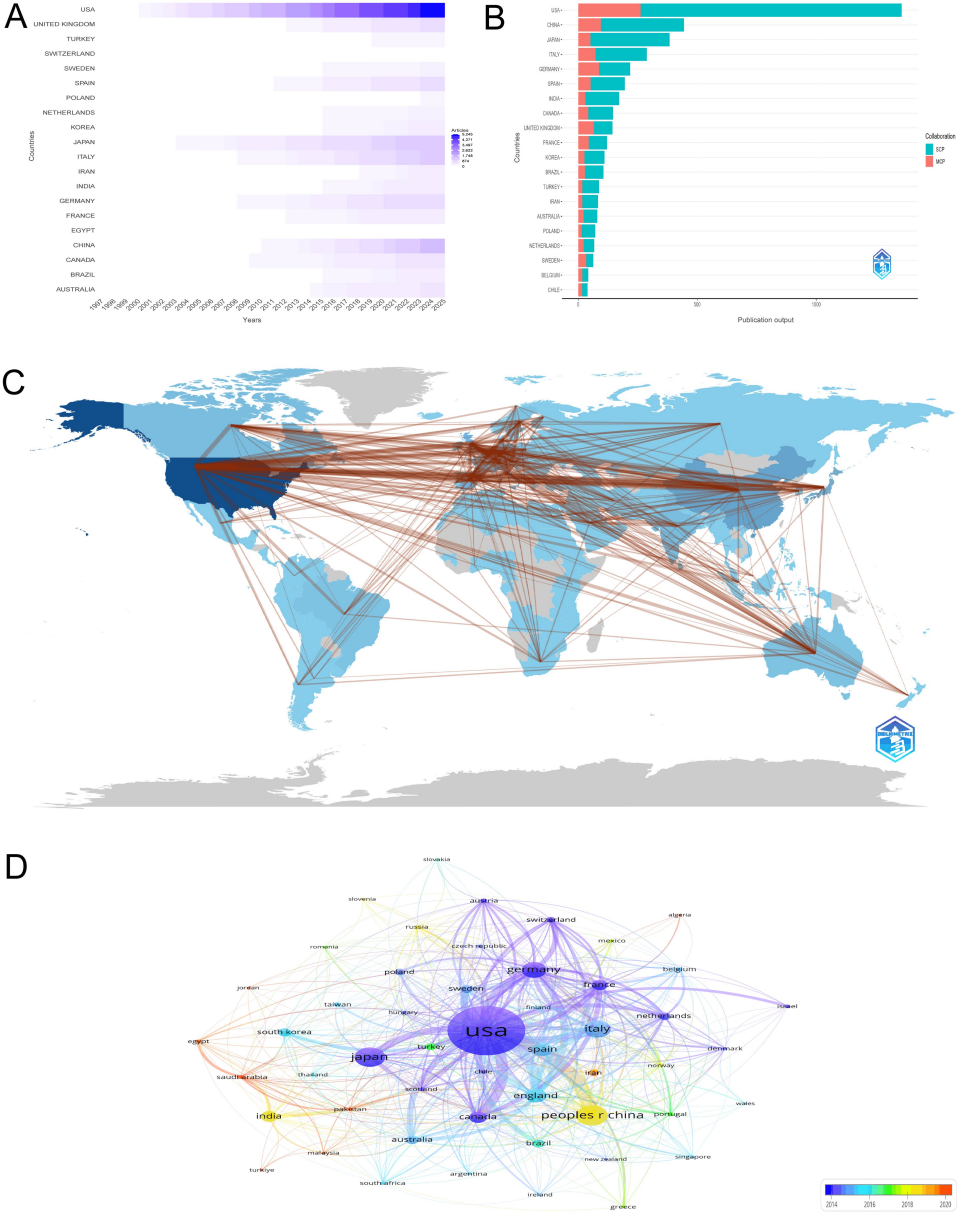
Global research contributions and collaboration networks. **(A)** Temporal trends in national research output, emphasizing the dominance of the United States (5,245 articles in 2025) and rapid growth in China (1,556 articles in 2025). The figure highlights disparities in research capacity between high-income and emerging economies. **(B)** Contributions of countries categorized by single-country (SCP) and multiple-country publications (MCP), highlighting the U.S. (1,359 articles, 19.3% MCP) and China (444 articles, 21.4% MCP) as collaborative hubs. The analysis underscores varying degrees of international partnership in advancing HIV-Alzheimer’s disease research. **(C)** Global collaboration network visualization, where node size indicates publication volume and link strength reflects collaborative partnerships. The U.S. serves as the central hub, with significant collaborations with China (96 joint articles), the U.K. (76), and Germany (64). This network emphasizes the interconnected nature of global research efforts. **(D)** Co-authorship network map, with node size proportional to publication volume and color gradient indicating recency. The U.S. (1,666 publications) and China (467 publications) emerge as dominant contributors, with warmer tones highlighting recent surges in Chinese research output.

The bibliometric analysis of 4,856 articles offers a comprehensive overview of the global research landscape on the intersection of HIV and AD. The United States has been a dominant force, with a steady increase in publications reaching 5,245 in 2025. Germany and Japan also play significant roles, contributing 865 and 1,182 articles, respectively, in 2025. Notably, China has shown a remarkable growth trajectory since 2000, publishing 1,556 articles in 2025. The United Kingdom, France, and Canada have maintained consistent growth, reaching 729, 471, and 585 articles, respectively, in 2025. This analysis highlights the collaborative and competitive nature of global research efforts. The visual representation in Figure 3A clearly depicts the global distribution and growth of research output over time in this dynamic field.

Figure 3B presents a detailed analysis of the most relevant countries by corresponding authors in the field of HIV and AD research, distinguishing between single-country publications (SCP) and multiple-country publications (MCP). The United States is the leading contributor with 1,359 articles, of which 19.3% are MCP, indicating a robust collaborative research environment. China follows with 444 articles, having a MCP percentage of 21.4%, reflecting its growing role in international research partnerships. Japan and Italy also make significant contributions, with 384 and 288 articles, respectively. Japan has a MCP percentage of 13.3%, while Italy’s MCP percentage is 25.3%, highlighting varying degrees of international collaboration. Germany and the United Kingdom further underscore the collaborative nature of this field, with Germany contributing 218 articles (39.9% MCP) and the United Kingdom 144 articles (45.8% MCP). This analysis illustrates the diverse levels of international collaboration and the prominent positions of certain countries in advancing research on the intersection of HIV and AD.

Figure 3C vividly illustrates the global collaboration network in the study of the intersection between HIV/AIDS and AD, emphasizing a substantial international research effort. The United States is a central hub within this network, characterized by an extensive number of collaborations. Notably, the USA has the most significant collaborations with China, totaling 96, highlighting a strong research partnership. Additionally, the USA maintains a robust collaborative relationship with the United Kingdom, with 76 joint efforts, and Germany, marked by 64 collaborations, indicating a key role in the field. The USA also connects with Italy (63 collaborations), Canada (61), Japan (50), and Sweden (41), showcasing a broad international engagement. These connections underscore the interconnected nature of research in this domain and the importance of international cooperation in advancing our understanding of the intersection between HIV/AIDS and AD. This global collaborative landscape is vital for fostering innovation and consolidating knowledge, essential for progress in the neuroscience field.

The visualization in Figure 3D offers a detailed representation of the co-authorship network among countries in the field of HIV/AIDS and AD research. The network map uses node sizes to indicate the volume of publications, where larger nodes represent a higher number of research outputs. The United States is the most prominent contributor with 1,666 publications, marking it as the central hub in this research domain. China, with 467 publications, is also highlighted, particularly with a warmer color indicating its significant and recent surge in research contributions. Japan and Italy follow with 447 and 373 publications respectively, and total link strengths of 166 and 290, underscoring their substantial research engagement. Germany, with 340 publications and a link strength of 388, and France, with 184 publications and a link strength of 196, also demonstrate considerable collaborative efforts and research influence within this domain. This co-authorship map effectively encapsulates the collaborative dynamics, emphasizing the pivotal contributions of major research hubs and the indispensable role of international collaboration in propelling scientific discovery in this critical area of neuroscience.

### 3.3 Institutional contributions to research

**Figure 4:**
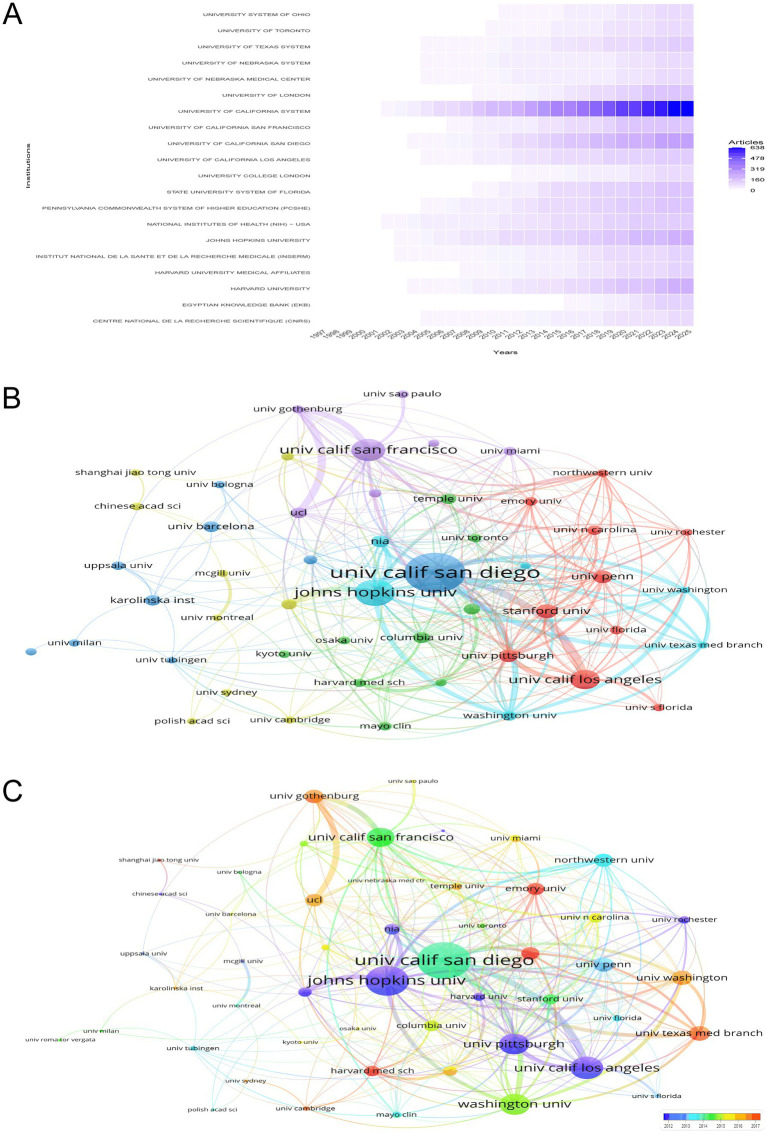
Institutional research contributions and collaborative networks. **(A)** Temporal trends in institutional research output, showcasing the prominence of the NIH (USA) and the University of California System. The NIH demonstrates a 2002–2025 surge, peaking at 143 articles in 2024, while the University of California System reaches 638 articles in 2025. **(B)** Co-authorship network map emphasizing publication counts, with the University of California San Diego (137 documents) and Johns Hopkins University (91 documents) as key contributors. **(C)** Co-authorship network incorporating total link strength and temporal trends, highlighting institutions like Emory University and Harvard Medical School (warmer tones) for their recent impactful contributions.

Figure 4A delineates the temporal trajectory of research contributions from various institutions in the study of the intersection between HIV and AD, highlighting the evolving landscape of scientific output. The National Institutes of Health (NIH)—USA and the University of California System stand out as prominent contributors. The NIH—USA has shown a steady increase in publication rates since the late 1990s, with a significant escalation in output from 2002 onwards, reaching a peak of 143 articles in 2024 and maintaining this level in 2025. The University of California System has also demonstrated a marked rise in its research output, with a notable surge from 2002 to 2025, culminating in 638 articles in 2025. The Egyptian Knowledge Bank (EKB), although starting with a modest contribution in 2005, has shown a significant increase in publications, particularly from 2019 onwards, with a total of 126 articles in 2025. These patterns underscore the dynamic and expanding nature of institutional involvement, reflecting the escalating global interest and investment in research at the nexus of HIV and AD.

Figures 4B,C offer a nuanced depiction of the co-authorship network among various institutions in the research domain of HIV/AIDS and AD. Figure 4B emphasizes publication counts, where institutions such as the University of California San Diego, with 137 documents, and Johns Hopkins University, with 91 documents, are prominently featured due to their substantial contributions. Figure 4C enriches this analysis by incorporating both total link strength and the temporal dimension of publications. Here, the University of California San Diego leads with a total link strength of 108, indicating extensive collaborative networks, closely followed by Johns Hopkins University with 88. Other significant contributors include the University of California San Francisco and the University of California Los Angeles, with total link strengths of 56 and 66, respectively. The color gradient in Figure 4C highlights institutions like Emory University, Harvard Medical School, and the University of Cambridge, which are represented in warmer tones, indicating their recent and impactful contributions. These institutions have total link strengths of 36, 31, and 13 respectively, underscoring their pivotal roles in current research dynamics. Additionally, institutions such as the University of Pennsylvania and Columbia University, with total link strengths of 41 and 32 respectively, are also notable for their collaborative efforts. These visualizations collectively highlight the collaborative landscape, emphasizing the importance of key institutions in advancing research at the intersection of HIV/AIDS and AD.

### 3.4 Journal-level analysis of research output

**Figure 5:**
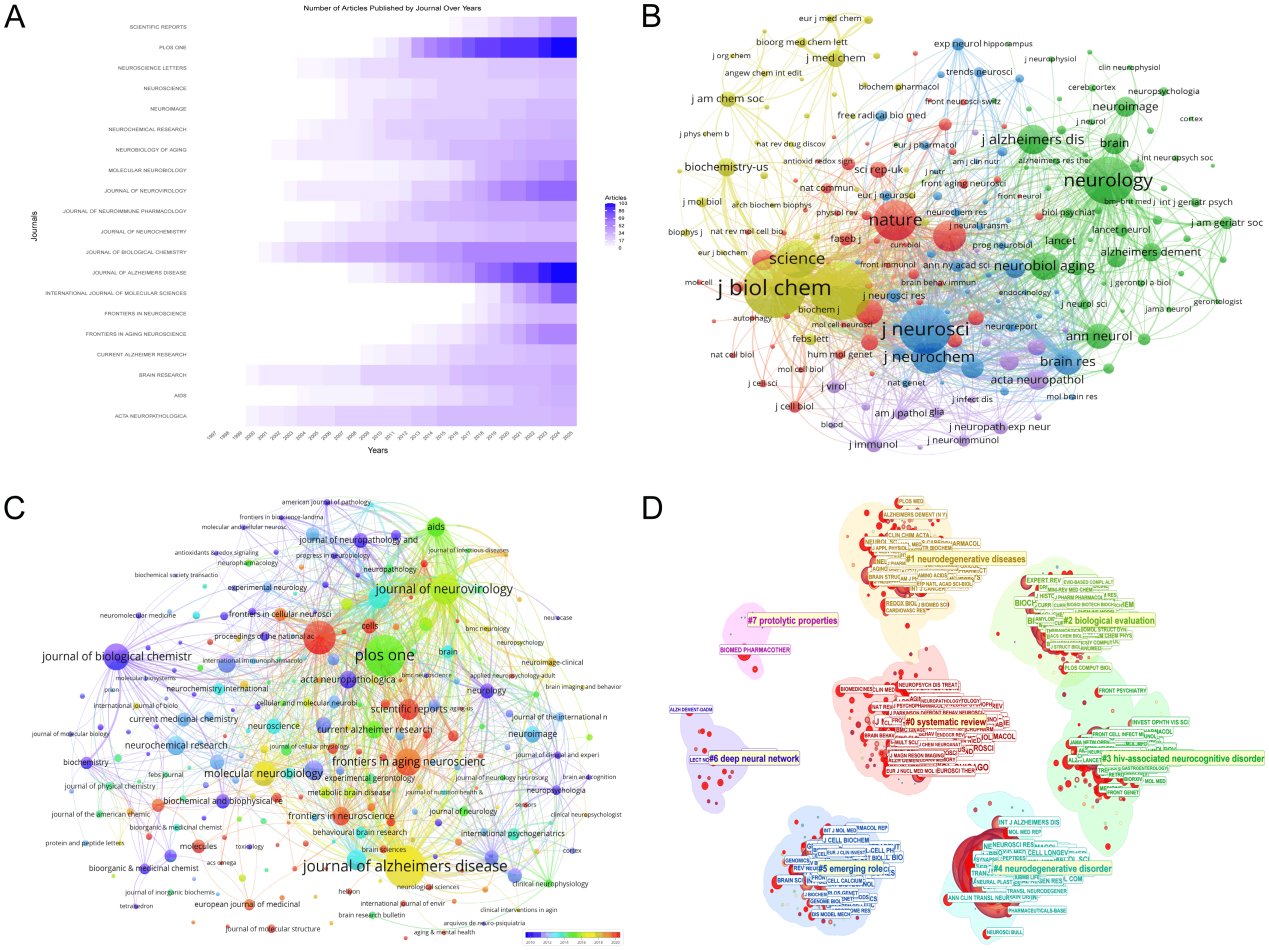
Journal-level analysis. **(A)** Temporal trends in journal publication output, emphasizing growth in journals such as *PLOS ONE* (102 articles in 2025) and *Journal of Alzheimer’s Disease* (103 articles in 2025). **(B)** Co-citation network of journals, with node size proportional to citation impact. *Journal of Biological Chemistry* (9,148 citations) and *PNAS* (8,945 citations) emerge as central nodes, underscoring their influence in shaping research discourse. **(C)** Journal coupling visualization, where node diameter reflects publication volume and color gradient indicates recency. *Journal of Alzheimer’s Disease* and *PLOS ONE* dominate, while journals like *Frontiers in Aging Neuroscience* (57 documents) highlight emerging trends. **(D)** Eight thematic clusters in HIV-Alzheimer’s research, highlighting key journals: *ARCH NEUROL-CHICAGO* (Cluster 0), *AM J CLIN NUTR* (Cluster 1), *J AM CHEM SOC* (Cluster 2), *J IMMUNOL* (Cluster 3), *J NEUROSCI* (Cluster 4), *J CELL BIOL* (Cluster 5), *IEEE ACCESS* (Cluster 6), and *BIOMED PHARMACOTHER* (Cluster 7).

The analysis of publication trends across key scientific journals, as shown in Figure 5A, reveals a substantial increase in research output from 1994 to 2025, focusing on the intersection of HIV/AIDS and AD. The *Journal of Biological Chemistry* has been a leading publisher with a total of 56 articles in 2025, marking a significant rise from only 3 articles in 2000. *PLOS ONE* has also shown remarkable growth, publishing 102 articles in 2025, up from none in 2007. The *International Journal of Molecular Sciences* has seen a substantial increase as well, with 76 articles in 2025 compared to none in 2008. The *Journal of Alzheimer’s Disease* has consistently published in this area, reaching 103 articles in 2025, up from 1 in 2005. These statistics highlight the increasing relevance and contribution of these journals to the scientific dialogue on HIV/AIDS and AD, reflecting the expanding interest and advancements in this critical area of research.

Figure 5B illustrates the co-citation journal network in the field of HIV/AIDS and AD research, emphasizing the intellectual structure and interconnectivity of pivotal journals. The “*Journal of Biological Chemistry*” emerges as a central node with 9,148 citations and a total link strength of 721,409, highlighting its significant influence. “*PNAS*” follows with 8,945 citations and 741,887 in total link strength, while “*Journal of Neuroscience*” has 7,107 citations and 631,463 in total link strength. “*Neurology*” is also prominent with 6,881 citations and 508,765 in total link strength. “*Nature*” and “*Science*” contribute substantially with 5,607 and 5,136 citations respectively, and total link strengths of 457,228 and 432,028. “*Journal of Neurochemistry*” and “*PLOS ONE*” are notable with 4,439 and 4,029 citations, and total link strengths of 453,886 and 283,535. “*Journal of Alzheimer’s Disease*” and “*Neurobiological Aging*” add significant contributions with 3,759 and 3,494 citations, and total link strengths of 272,011 and 282,034. These journals play a crucial role in shaping the research landscape, as depicted in Figure 5B.

Figure 5C presents the network of Journal Coupling in the research area of HIV/AIDS and AD, visualized using VOSviewer. The diameter of each node corresponds to the number of publications, with larger nodes indicating higher publication volumes. The “*Journal of Alzheimer’s Disease*” and “*PLOS ONE*” are particularly prominent, with 103 and 102 documents respectively, reflecting their significant contributions to the field. The “*International Journal of Molecular Sciences*” also shows a substantial publication count with 76 documents. The color gradient, with warmer tones indicating more recent activity, highlights journals such as “*Frontiers in Aging Neuroscience*” and “*Molecular Neurobiology*,” which have 57 and 50 documents respectively, suggesting their growing influence. The “*Journal of Biological Chemistry*” stands out with a total link strength of 8,520, indicating its extensive co-citation connections and central role in the field. Other notable journals include “*Journal of Neuroimmune Pharmacology*” and “*Neurobiology of Aging*,” with total link strengths of 19,437 and 9,640 respectively, underscoring their importance in current research discourse. This visualization effectively captures the key journals shaping the research landscape, emphasizing their roles in advancing our understanding of the intersection between HIV/AIDS and AD.

The journal co-citation clustering analysis (Figure 5D) identified eight distinct thematic clusters, each characterized by unique research focuses and contributions from leading journals. Cluster 0 (Size = 235, Silhouette = 0.738), with the highest silhouette score among large clusters, centered on systematic reviews and cognitive dysfunction in neurodegenerative diseases, led by *ARCH NEUROL-CHICAGO* (Degree = 73). Cluster 1 (Size = 183, Silhouette = 0.554) emphasized oxidative stress mechanisms in HAND, with *AM J CLIN NUTR* (Degree = 71) as its top contributor. Cluster 2 (Size = 174, Silhouette = 0.768) focused on amyloid fibril characterization and molecular docking studies, dominated by *J AM CHEM SOC* (Degree = 73). Cluster 3 (Size = 141, Silhouette = 0.713) explored cerebrospinal fluid biomarkers in HIV-1 encephalitis, with *J IMMUNOL* (Degree = 93) demonstrating the highest connectivity. Cluster 4 (Size = 131, Silhouette = 0.879), the most cohesive cluster, examined endoplasmic reticulum stress in Alzheimer’s pathogenesis, led by *J NEUROSCI* (Degree = 108). Cluster 5 (Size = 128, Silhouette = 0.654) investigated microglial activation in neuroinflammatory pathways, with *J CELL BIOL* (Degree = 88) as its core journal. Cluster 6 (Size = 19, Silhouette = 0.991) represented computational approaches using deep neural networks for Alzheimer’s diagnosis, led by *IEEE ACCESS* (Degree = 58). Lastly, Cluster 7 (Size = 10, Silhouette = 0.992) focused on proteolytic mechanisms in neurodegeneration, with *BIOMED PHARMACOTHER* (Degree = 36) as its key contributor. These clusters highlight the interdisciplinary landscape of HIV-Alzheimer’s research, spanning clinical neurology, molecular biology, and computational science, with influential journals driving advancements in each subfield.

### 3.5 Author-level analysis of research contributions

**Figure 6:**
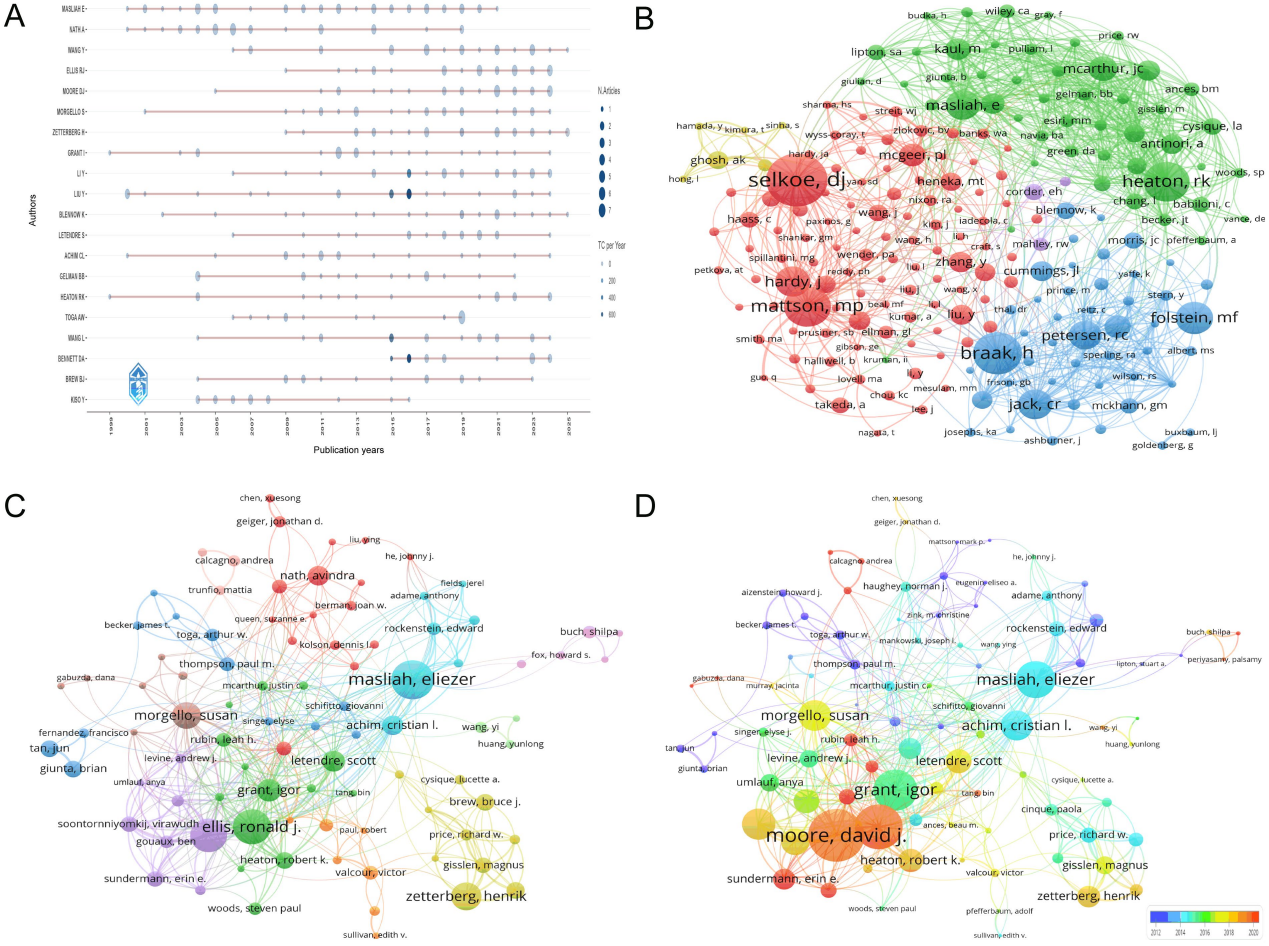
Author-level contributions and networks. **(A)** Temporal distribution of author productivity, highlighting sustained contributors (e.g., MASLIAH E, NATH A) with consistent annual publications and emerging researchers (e.g., WANG Y) demonstrating post-2015 growth. **(B)** Co-citation network of authors, with node size reflecting academic influence. Prominent clusters around Selkoe, DJ (509 citations) and Mattson, MP (390 citations) highlight focal areas of research intensity. **(C)** Co-authorship network map, illustrating collaborative hubs such as Masliah, E (28 documents, 1,396 citations) and Ellis, RJ (25 documents, 363 citations). Line thickness indicates collaborative extent, emphasizing knowledge integration across disciplines. **(D)** Author influence visualization, ranked by total link strength and recency. Moore, DJ (total link strength = 113) and Ellis, RJ (96) emerge as central figures, while warmer tones highlight recent contributors like Sundermann, EE.

Figure 6A presents the temporal distribution of authors’ publication outputs in the HIV-Alzheimer’s research field, generated using the bibliometric R package to visualize productivity trends. The visualization highlights sustained contributions from authors such as MASLIAH E and NATH A, whose consistent publication records (e.g., MASLIAH E maintaining ≥1 paper/year from 2000 to 2021) reflect longstanding leadership in neuropathological studies at the HIV-Alzheimer’s interface. Emerging contributors like WANG Y demonstrate exponential growth post-2015, with a peak of 4 publications in 2023, suggesting rising research momentum in translational approaches. Notable spikes include BENNETT DA’s 2015–2016 surge (3 papers) and TOGA AW’s 2018–2019 productivity (8 papers), likely driven by advances in neuroimaging and biomarker discovery. The analysis also identifies collaborative hubs, such as ZETTERBERG H and BLENNOW K, whose overlapping publication timelines (e.g., 2019–2023) correlate with breakthroughs in cerebrospinal fluid biomarker development. Collectively, Figure 6A underscores the field’s evolution from early pathological investigations led by MASLIAH E and NATH A to contemporary multi-disciplinary efforts spearheaded by WANG Y and TOGA AW, providing critical insights into researcher influence and emerging trends for future collaborative opportunities.

Figure 6B presents a comprehensive visualization of co-citation author networks in the interdisciplinary field of HIV and AD research, generated through VOSviewer’s network analysis capabilities. The node size is proportional to the citation count, reflecting each author’s academic influence. Prominent clusters, such as those around Selkoe, DJ (509 citations, total link strength 4,145), and Mattson, MP (390 citations, total link strength 4,506), are clearly visible, underscoring their central roles and extensive impact within this research community. These clusters reveal concentrated research interests and frequent co-citation patterns, indicating areas of intense scholarly focus. The network’s highly interconnected structure, with its dense web of connections, highlights the collaborative nature of research in this field, where insights from various disciplines converge to advance understanding. Authors like Heaton, RK (384 citations, total link strength 4,815), and Petersen, RC (277 citations, total link strength 2,670), are also notable for their high citation counts and central positions within the network, demonstrating their significant contributions to knowledge advancement in this domain.

Figure 6C, crafted with VOSviewer, presents a comprehensive co-authorship network map that captures the collaborative dynamics in the field of HIV/AIDS and AD research. The map uses node sizes to represent the document count of each author, thereby highlighting their publication contributions, while the line thicknesses between nodes indicate the extent of their collaborations. Prominent figures such as Masliah, Eliezer, with 28 documents and 1,396 citations, Ellis, Ronald J., with 25 documents and 363 citations, and Moore, David J., with 24 documents and 374 citations, stand out for their significant contributions. Additionally, Zetterberg, Henrik, Morgello, Susan, and Grant, Igor are recognized for their substantial document counts and high citation counts, respectively, emphasizing their influential roles in shaping the understanding of the intersection between HIV/AIDS and AD through their collaborative efforts. Figure 6D further enriches this view by focusing on the authors’ influence as measured by total link strength, where Moore, David J. leads with a total link strength of 113, indicating his central role in the research community. Ellis, Ronald J., and Babiloni, Claudio, follow with total link strengths of 96 and 92, respectively, underscoring their substantial contributions. The network also identifies authors who have been particularly active in recent years, as indicated by warmer color tones, such as Sundermann, Erin E., and Rubin, Leah H., suggesting their growing influence. This visual representation underscores the principal contributors to the research landscape and captures the dynamic evolution of collaborative efforts, offering a snapshot of the contemporary research dynamics at the intersection of HIV/AIDS and AD.

### 3.6 Document-level analysis of research impact

**Figure 7:**
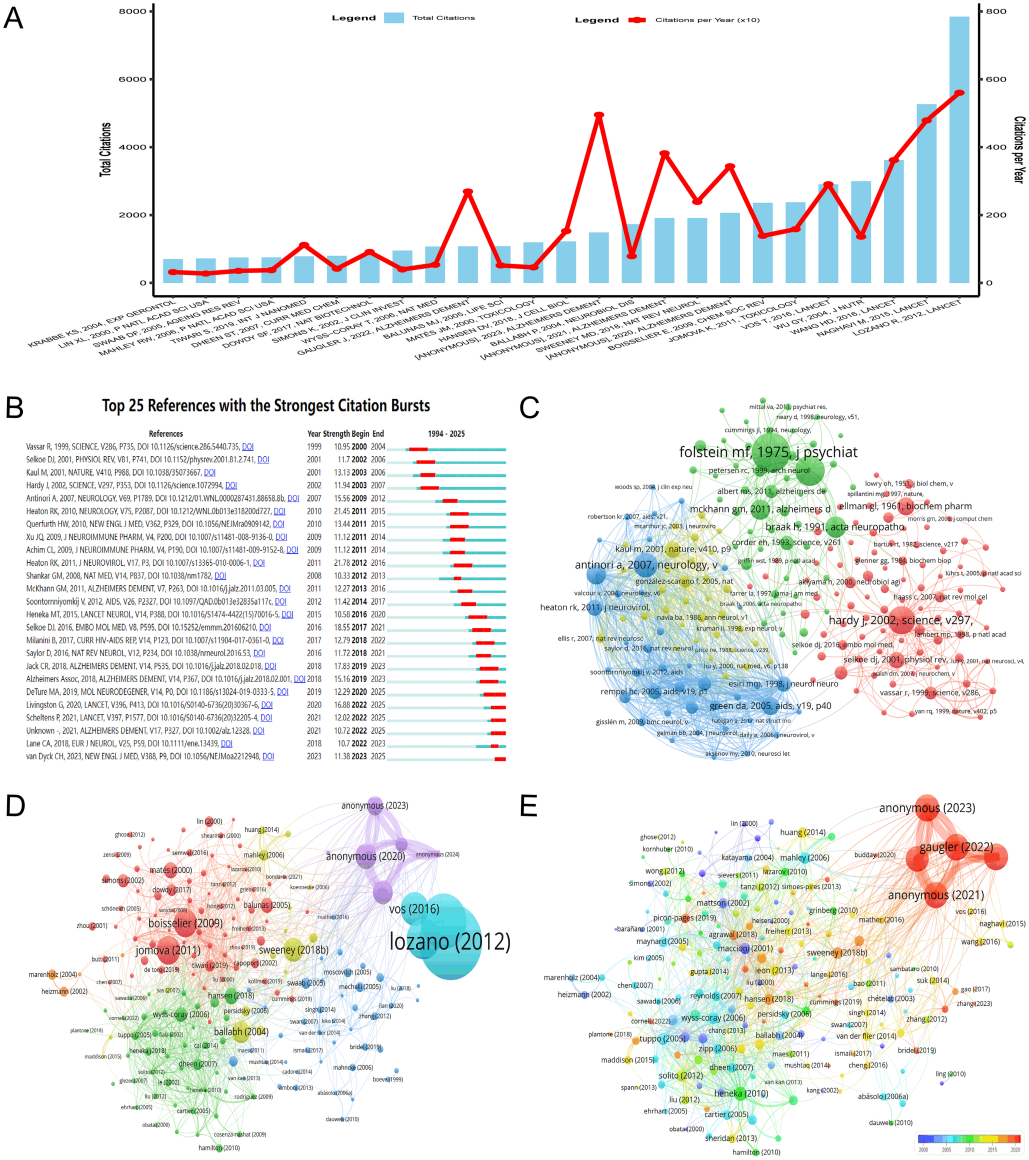
Document-Level Impact and Citation Dynamics. **(A)** Most globally cited documents, ranked by total citations and citations per year. Lozano et al. (17, 7,845 citations) and Naghavi et al. (18, 5,266 citations) are highlighted for their foundational contributions to epidemiological and pathological overlaps between HIV and AD. **(B)** Temporal citation bursts identifying pivotal studies driving research forward. Early works (21, 22) established molecular links, while recent studies (29) focus on therapeutic strategies. **(C)** Co-citation network clusters representing foundational works (31, 35) and molecular mechanisms (33). The network underscores the interconnectedness of historical and contemporary research directions. **(D)** Bibliographic coupling visualization highlighting key documents shaping the field. Lozano’s 2012 work (7,845 citations) and Anonymous’s 2023 study (total link strength = 2,181) reflect the coexistence of foundational and emerging contributions. **(E)** Temporal network incorporating total link strength and publication year. Sweeney’s 2018 work (1,911 citations) persists as a central node, while recent entries (35) suggest evolving research priorities.

The bibliometric analysis of the most globally cited documents in the field of HIV/AIDS and AD intersection, as depicted in Figure 7A, reveals several seminal papers that have significantly advanced our understanding of this complex relationship. Lozano et al. (17) published a highly influential paper in *The Lancet*, which has garnered 7,845 citations. This work has likely provided critical insights into the global burden of diseases, including the epidemiological links between HIV/AIDS and neurodegenerative conditions such as AD. Naghavi et al. (18) further contributed to this field with their *Lancet* publication, amassing 5,266 citations. Wang et al. (19) added to this body of knowledge with their *Lancet* publication, which has accumulated 3,618 citations, potentially highlighting the clinical or pathological overlaps between HAND and AD. The paper by Anonymous (20) published in *Alzheimer’s & Dementia* stands out with a total of 1,486 citations and a particularly high TC per Year of 495.33, indicating its significant and recent impact on the field. These highlighted documents, characterized by their high total citations and notable TC per Year values, have not only been frequently cited but have also consistently influenced the research landscape, driving forward the frontiers of knowledge in this interdisciplinary area.

The analysis of significant citation bursts in the literature on the intersection of HIV/AIDS and AD, as visualized in Figure 7B, reveals key trends and contributions over time. The citation bursts identified in the dataset highlight pivotal studies that have driven research forward in this field. Early studies, such as Vassar et al. (21) and Selkoe (22), laid foundational insights into the molecular mechanisms linking HIV and neurodegenerative processes, particularly regarding beta-amyloid pathology. These works established critical links between HIV-associated neuroinflammation and Alzheimer’s-like pathologies, setting the stage for subsequent research. From 2000 to 2010, studies such as Hardy et al. (23) and Kaul et al. (24) further explored the role of neuroinflammation and immune dysregulation in HIV-related neurological disorders, emphasizing the importance of understanding how HIV impacts brain health. Notably, Antinori et al. (25) and Heaton et al. (26) contributed significantly to understanding HAND and their overlap with AD pathology, particularly in the context of aging populations with HIV. In the 2010s, research shifted toward more specific mechanisms, with studies like Shankar et al. (27) and Heneka et al. (28) investigating the role of tau protein and beta-amyloid in HIV-related neurodegeneration. These works highlighted the convergence of HIV and AD in terms of shared pathological features, such as neuroinflammation and protein aggregation. More recent studies, such as those by Livingston et al. (29) and van Dyck et al. (30), have focused on therapeutic strategies and the long-term neurological consequences of HIV infection, particularly in the context of aging. These contributions underscore the evolving nature of research in this field, moving from descriptive pathology to mechanistic insights and potential interventions. Overall, the citation bursts identified in Figure 7B reflect the dynamic progression of research at the intersection of HIV/AIDS and AD, with each phase of study building on prior findings to advance understanding of this complex relationship.

The co-citation network visualization presented in Figure 7C delineates the academic influence within the literature on the intersection of HIV/AIDS and AD. The network is organized into distinct clusters, each representing a significant area of focus. The green cluster, which is the most prominent, includes foundational works such as Folstein et al. (31) from “*Journal of Psychiatric Research*” with 300 citations and a total link strength of 649, indicating a substantial impact on the field. This cluster also features influential works like Braak and Braak (32) in “*Acta Neuropathologica*” with 140 citations and a total link strength of 417, underscoring their significant contributions to early understandings of AD pathology. The red cluster, though less interconnected, highlights key studies such as Hardy and Selkoe (33) in “*Science*” with 197 citations and a total link strength of 746, marking important contributions to the understanding of molecular mechanisms in neurodegenerative diseases. The blue cluster, while less prominent, contains significant works like Antinori et al. (25) in “*Neurology*” with 169 citations, indicating a focus on the neurological complications of HIV. The yellow cluster, though the least interconnected, includes foundational works such as McKhann et al. (34) in “*Neurology*” with 218 citations and a total link strength of 608, marking important contributions to the clinical diagnosis of AD. This analysis captures the key literature that has been driving research at the intersection of HIV/AIDS and AD, highlighting the interconnectedness of these fields and the influence of seminal works on contemporary research directions.

The bibliometric analysis, as depicted in Figures 7D,E, reveals the pivotal documents that have shaped the research landscape at the intersection of HIV/AIDS and AD. In Figure 7D, the visualization of bibliographic coupling highlights several key documents. Lozano’s 2012 work stands out as particularly influential, with an extraordinary 7,845 citations and a total link strength of 25, indicating its foundational role in the field. This is further underscored by Naghavi’s 2015 study, which has garnered 5,266 citations and a total link strength of 40, marking it as another seminal contribution. Additionally, Wang’s 2016 study, with 3,618 citations and a total link strength of 43, and Wu’s 2004 work have significantly contributed to the discourse with 2,998 citations. These documents collectively illustrate the depth and breadth of research that has been foundational in understanding the complex interplay between HIV/AIDS and AD. Figure 7E complements this analysis by incorporating the temporal dimension, where node sizes represent total link strength and the color gradient indicates the publication year. The document by Anonymous from 2023, with a total link strength of 2,181, emerges as a recent key contributor, while Gauger’s 2022 work, with a total link strength of 2,127, signifies another contemporary piece of scholarship gaining traction. The presence of documents like Anonymous (35, 36) in Figure 7E, depicted in warmer tones, suggests their rising importance in the field, highlighting the ongoing evolution of research priorities. The recurring mention of Sweeney’s 2018 work across both figures underscores its enduring relevance, with a citation count of 1,911 and a total link strength of 134 in Figure 7D, and its continued significance in the network depicted in Figure 7E. These visualizations collectively capture the dynamic and evolving landscape of research at the nexus of HIV/AIDS and AD, where foundational studies coexist with and inform emerging insights, collectively propelling the field forward.

### 3.7 Thematic evolution and research trends

**Figure 8:**
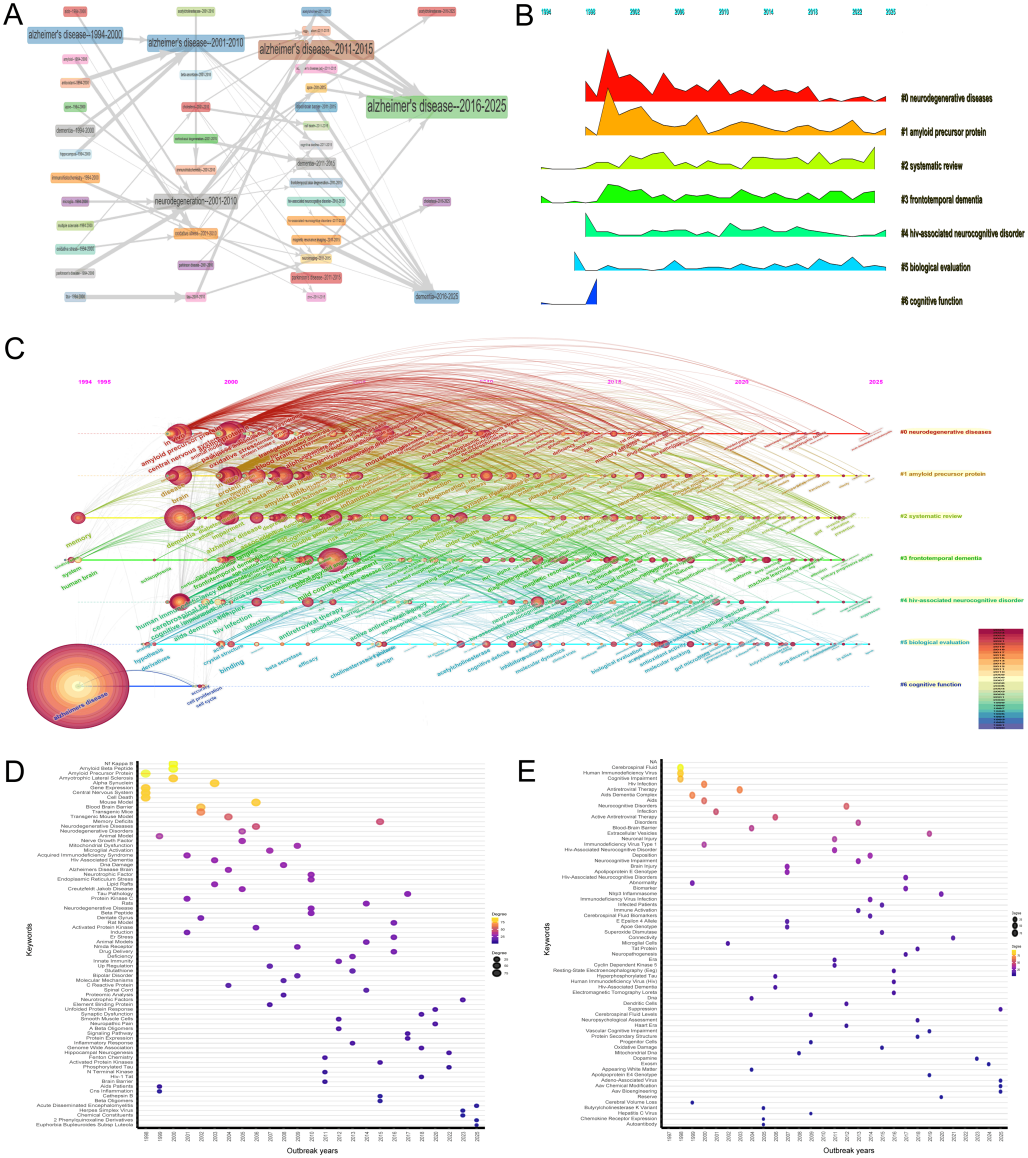
Thematic evolution and research trends. **(A)** Temporal trends in research themes (1994–2025), transitioning from descriptive pathology (“Alzheimer’s disease,” “AIDS”) to mechanistic insights (“blood–brain barrier,” “MRI”) and recent focus on cholesterol metabolism. The figure reflects the maturation of research toward integrative approaches. **(B)** Mountain plot illustrating keyword clusters and their peak periods. The “neurodegenerative diseases” cluster (Cluster 0) peaked in the early 2000s, while “HIV-associated neurocognitive disorder (HAND)” (Cluster 4) gained prominence post-2010, reflecting shifts in research emphasis. **(C)** Timeline visualization of keyword clustering, showing progression from broad neurodegenerative themes to specific investigations into amyloid pathology and biomarkers. Recent trends highlight translational applications of machine learning. **(D)** Temporal analysis of the “neurodegenerative diseases” cluster (Cluster 0) from 1997 to 2025, highlighting top three keywords for each year: 1997–1999 (“central nervous system,” “gene expression,” “cell death”); 2000–2009 (“amyotrophic lateral sclerosis,” “oxidative stress,” “transgenic mice”); 2010–2019 (“neurotrophic factor,” “endoplasmic reticulum stress,” “synaptic plasticity”); 2020–2025 (“neuropathic pain,” “hippocampal neurogenesis,” “protein misfolding”). **(E)** Temporal trends in research foci within Cluster 4 HAND from 1997 to 2025, highlighting the top three keywords for each year: “cerebrospinal fluid,” “human immunodeficiency virus,” and “cognitive impairment” (1998–1999); “antiretroviral therapy,” “blood–brain barrier,” and “microglial cells” (2000–2009); “neuronal injury,” “HAND,” and “cyclin-dependent kinase 5” (2010–2019); and “nlrp3 inflammasome,” “adeno-associated virus,” and “aav bioengineering” (2020–2025).

Figure 8A provides a comprehensive overview of the thematic evolution in the research trends at the intersection of HIV/AIDS and AD from 1994 to 2025. Initially, from 1994 to 2000, foundational themes such as “Alzheimer’s disease,” “AIDS,” and “Amyloid” were predominant. The period from 2001 to 2010 expanded into areas like “Neurodegeneration,” “Oxidative Stress,” and “Parkinson’s Disease,” indicating a growing recognition of the interconnectedness of these conditions. By the 2011–2015 period, there was a notable emphasis on “Blood–Brain Barrier,” “Dementia,” and “MRI,” reflecting an increasing focus on the mechanisms underlying neurodegenerative processes and the role of imaging in diagnosis. In the recent period from 2016 to 2025, themes like “Alzheimer’s disease,” “Dementia,” and “Cholesterol” have gained prominence, highlighting a concentrated research interest in understanding the complexities and interrelations of these conditions. The interconnectedness of these themes illustrates the complex and multifaceted nature of research in this field, emphasizing the importance of a holistic approach to studying the interactions between HIV/AIDS and AD.

The mountain plot (Figure 8B) and keyword clustering with timeline visualization (Figure 8C) generated by CiteSpace offer a comprehensive and detailed analysis of the research trends and thematic evolution in the intersection of HIV/AIDS and AD research from 1994 to 2025. The mountain plot highlights several key clusters and their respective peaks, indicating periods of heightened research interest. For instance, the “neurodegenerative diseases” cluster (Cluster 0) shows a significant peak in the early 2000s, reflecting a surge in research interest during that period, while the “amyloid precursor protein” cluster (Cluster 1) peaks around 2002, suggesting a focused research effort on the role of amyloid proteins in neurodegenerative processes, particularly in the context of HAND. Other clusters such as “systematic review” (Cluster 2), “frontotemporal dementia” (Cluster 3), “HIV-associated neurocognitive disorder” (Cluster 4), “biological evaluation” (Cluster 5), and “cognitive function” (Cluster 6) also exhibit distinct peaks and trends over time, indicating shifts in research focus. The keyword clustering and timeline visualization in Figure 8C further corroborate and expand on these trends, revealing a clear progression from general to specific areas of study over time. This progression reflects the maturation and specialization of research in this field, with broader themes such as neurodegenerative diseases and amyloid precursor proteins dominating the early research landscape, while more targeted investigations into HAND and biological mechanisms emerging in later years. By the final phase of the timeline, from 2016 to 2025, the research has expanded to encompass a wide array of implications, indicating a rich, multifaceted understanding of these conditions. Overall, these visualizations provide a detailed and nuanced picture of the evolving research landscape at the intersection of HIV/AIDS and AD, highlighting the shifting priorities and increasing depth and breadth of investigation over the past three decades.

The temporal analysis of the “neurodegenerative diseases” cluster (Cluster 0) from 1997 to 2025, as depicted in Figure 8D, reveals distinct trends and evolving research focuses across four key periods. In the initial period (1997–1999), research was centered on foundational aspects such as “central nervous system,” “gene expression,” and “cell death,” with early exploration of “amyloid precursor protein” and “multiple sclerosis.” During the second period (2000–2009), the focus expanded to include specific diseases like “amyotrophic lateral sclerosis” and mechanisms such as “oxidative stress” and “nitric oxide,” with the introduction of “transgenic mice” and investigation of the “blood–brain barrier.” The third period (2010–2019) saw diversification into broader themes like “neurotrophic factor” and “endoplasmic reticulum stress,” and the integration of genetic and synaptic research. The most recent period (2020–2025) has seen increased interest in areas such as “neuropathic pain,” “hippocampal neurogenesis,” and protein misfolding phenomena.

In the bibliometric analysis of the “HIV-associated neurocognitive disorder” cluster (Cluster 4) spanning from 1997 to 2025, as depicted in Figure 8E, distinct temporal shifts in research foci were evident. Between 1998 and 1999, foundational studies were predominant in the literature. Key terms such as “cerebrospinal fluid” (degree = 94), “human immunodeficiency virus” (degree = 86), and “cognitive impairment” (degree = 77) highlighted early efforts to characterize HIV-related neurological dysfunction. From 2000 to 2009, research expanded to encompass broader aspects of HIV neuropathology. Terms like “antiretroviral therapy” (degree = 70), “blood–brain barrier” (degree = 42), and “microglial cells” (degree = 10) emerged as central themes. Notably, terms such as “HIV-associated dementia” (degree = 8) and “hyperphosphorylated tau” (degree = 8) began to appear during this period, underscoring the growing recognition of overlaps between HAND and Alzheimer’s pathology. Between 2010 and 2019, research delved into detailed mechanistic studies and diagnostic advancements. Keywords like “neuronal injury” (degree = 37), “HIV-associated neurocognitive disorder” (degree = 31), and “cyclin-dependent kinase 5” (degree = 9) reflected a shift toward understanding cellular and molecular pathways. From 2020 to 2025, the focus shifted to emerging topics such as inflammatory pathways and novel therapeutic targets. Terms like “nlrp3 inflammasome” (degree = 19) indicated ongoing efforts to uncover new mechanisms. The appearance of “adeno-associated virus” (degree = 4) and “aav bioengineering” (degree = 4) in recent years suggested innovative approaches to gene therapy and neuroprotection.

### 3.8 Keyword analysis and research focus

**Figure 9:**
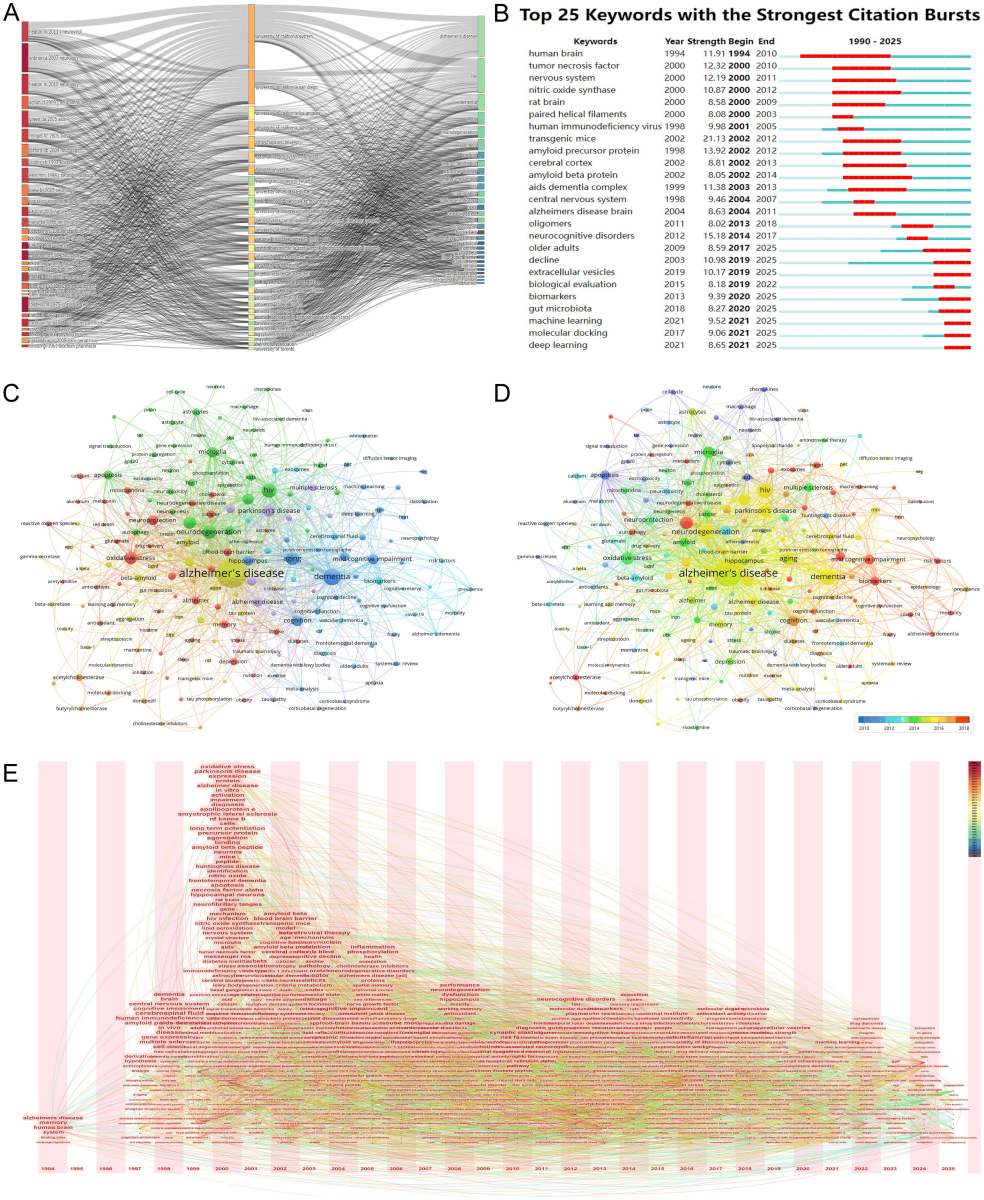
Keyword analysis and research focus. **(A)** Three-field plot linking key literature, institutions, and keywords. Influential works (e.g., “heaton rk 2011 *neurovirol*”) are associated with institutions like the University of California System and keywords such as “neuroinflammation” and “blood–brain barrier,” underscoring interdisciplinary knowledge integration. **(B)** Keyword burst detection chart highlighting emerging trends. Early bursts in “amyloid precursor protein” (2002–2012) transition to recent surges in “machine learning” (2021–2025), reflecting methodological advancements and data-driven research priorities. **(C)** Keyword co-occurrence network, with node size reflecting frequency. Central terms like “neuroinflammation” (154 occurrences) and “microglia” (115 occurrences) anchor the network, while secondary hubs such as “tau protein” and “oxidative stress” highlight pathological interfaces. **(D)** Keyword co-occurrence network with node size indicating total link strength and color gradient reflecting recency. Emerging terms like “deep learning” (27 occurrences) and “extracellular vesicles” (26 occurrences) signal translational and technological shifts in research focus. **(E)** Temporal evolution of keyword centrality (1994–2025), illustrating shifts from descriptive terms (“memory,” “Alzheimer’s disease”) to mechanistic themes (“amyloid beta peptide,” “apoptosis”) and recent emphasis on neuroinflammation and computational methods. The figure underscores the dynamic prioritization of research questions over time.

The Three-Field Plot (Figure 9A), generated using the bibliometrix R package, provides a comprehensive visualization of the intricate relationships among key literature, institutions, and keywords in the intersection of HIV/AIDS and AD research. The plot highlights several influential literature pieces, such as “heaton rk 2011 *neurovirol*,” “antinori a 2007 *neurology*,” and “green da 2005 *aids*,” which have significantly contributed to the understanding of neurodegenerative processes in the context of HIV and AD. These works are closely associated with prominent institutions like the University of California System, Johns Hopkins University, and Harvard University, indicating their pivotal role in advancing research in this domain. The visualization also emphasizes keywords such as “Alzheimer’s disease,” “HIV,” “dementia,” “neurodegeneration,” and “amyloid,” which are central to current research themes. Notably, terms like “neuroinflammation,” “cognitive impairment,” and “blood–brain barrier” emerge as significant areas of focus, reflecting the growing interest in understanding the complex interplay between HIV and neurodegenerative pathologies. The plot further reveals the dynamic nature of research collaborations and knowledge exchange, underscoring the importance of interdisciplinary efforts in addressing the multifaceted challenges at the intersection of HIV/AIDS and AD. This visualization effectively captures the evolving research landscape, highlighting both foundational contributions and emerging trends that continue to shape the field.

Figure 9B displays a Keyword Burst Detection chart, which vividly visualizes the top 25 keywords with the strongest citation bursts in the research area relevant to HIV/AIDS and AD from 1990 to 2025. This chart is crucial for discerning the trends and changes in research focus over time. Early on, keywords like “human brain” (burst strength 11.91, 1994–2010) and “nervous system” (burst strength 12.19, 2000–2011) showed significant bursts, highlighting the fundamental research on the neural aspects related to these diseases. As research progressed, terms such as “amyloid precursor protein” (burst strength 13.92, 2002–2012) and “amyloid beta protein” (burst strength 8.05, 2002–2014) emerged with strong bursts, in line with the exploration of AD’s pathological mechanisms involving amyloid-related pathways. More recently, keywords like “machine learning” (burst strength 9.52, 2021–2025) and “deep learning” (burst strength 8.65, 2021–2025) have shown strong citation bursts, suggesting an increasing trend of applying advanced computational methods to study the intersection of HIV/AIDS and AD. This indicates a shift towards more data-driven and innovative research approaches in understanding the complex pathological mechanisms of these diseases.

The co-occurrence network visualization in Figures 9C,D offers a comprehensive representation of author keywords in the HIV/AIDS-Alzheimer’s disease intersection field, addressing the need for broader keyword inclusion. Figure 9C, where node size reflects keyword frequency, reveals “Alzheimer’s disease” (1,101 occurrences, total link strength 1981) as the most central term, closely followed by “HIV” (224 occurrences, 527 link strength) and “neurodegeneration” (190 occurrences, 478 link strength). These nodes anchor the network, highlighting their foundational roles in bridging viral pathogenesis and neurodegenerative mechanisms. Expanding beyond the previously emphasized terms, Figure 9C also underscores secondary hubs such as “tau protein” (31 occurrences, 48 link strength), “beta-amyloid” (58 occurrences, 128 link strength), and “oxidative stress” (149 occurrences, 344 link strength), which are critical to Alzheimer’s pathological pathways. Meanwhile, Figure 9D, with node size indicating total link strength and warm hues signaling recency, emphasizes emerging trends like “machine learning” (26 occurrences, 41 link strength) and “deep learning” (27 occurrences, 27 link strength), reflecting methodological advancements. Notably, keywords such as “neuroinflammation” (154 occurrences, 362 link strength) and “microglia” (115 occurrences, 292 link strength) appear prominently in both figures, underscoring their role in mediating HIV-associated neurotoxicity and Alzheimer’s pathogenesis. The inclusion of terms like “blood–brain barrier” (59 occurrences, 107 link strength) and “amyloid precursor protein” (32 occurrences, 65 link strength) further illustrates the field’s focus on pathological interfaces between viral infection and amyloidopathy. This expanded analysis ensures a more nuanced understanding of the thematic landscape while adhering to bibliometric rigor and journal requirements.

The bibliometric analysis, as illustrated in Figure 9E, offers a detailed examination of the evolving research landscape at the intersection of HIV/AIDS and AD from 1994 to 2025. Initially, the research was centered around general cognitive and brain-related studies, with keywords such as “memory” and “Alzheimer’s disease” emerging in 1994, indicating an early focus on these areas. The centrality values of these keywords were relatively modest, suggesting that they were part of a larger research framework. Moving into the late 1990s, there was a shift towards more specific biological mechanisms, with terms like “cerebrospinal fluid” and “amyloid precursor protein” becoming more prominent, reflecting a deeper dive into the pathological processes underlying these conditions. The turn of the millennium marked a significant expansion in research themes, with a notable increase in the centrality of keywords like “amyloid beta peptide” and “apoptosis,” indicating a growing focus on cellular and molecular pathways in neurodegenerative diseases. The early 2000s continued this trend with a diversification of research interests, including a notable focus on “antiretroviral therapy” and “tau protein,” suggesting an emerging understanding of the interplay between viral infections and neurodegenerative processes. The mid-2000s saw a consolidation and specialization of research areas, with a focus on “mild cognitive impairment” and “neurodegenerative disorders,” and a rise in the centrality of therapeutic interventions and disease mechanisms. In recent years, the research has evolved to include more sophisticated and interdisciplinary approaches, as indicated by the emergence of keywords such as “neuroinflammation,” “machine learning,” and “extracellular vesicles,” reflecting a shift towards integrating advanced technologies and methodologies to better understand and address the complex interactions between HIV/AIDS and AD. This analysis underscores the dynamic nature of scientific inquiry and provides valuable insights into the future directions of this field, highlighting the continuous evolution and refinement of research interests over time.

## 4 Discussion

This bibliometric analysis provides a comprehensive overview of the research landscape at the intersection of HIV and AD. By examining 4,856 articles and reviews from the Web of Science Core Collection, we identified key trends in publication output, international collaboration, institutional contributions, journal dynamics, author networks, and thematic evolution. These findings highlight the growing academic interest in understanding the complex relationship between HIV and AD, particularly in terms of shared pathological mechanisms such as neuroinflammation, protein aggregation, and blood–brain barrier disruption (37). Key findings include a steady increase in publications, significant contributions from the United States and other high-income countries, and a shift in research focus from descriptive pathology to mechanistic insights and therapeutic strategies.

The rapid growth and global collaborative nature of the research reflect increasing recognition of HIV-AD interactions as a critical area of neuroscience. The transition from descriptive to mechanistic research indicates a maturing field, with implications for developing targeted therapies and diagnostic tools. The prominence of high-income countries and well-funded institutions underscores the resource-intensive nature of this research, while the focus on shared pathological mechanisms provides a foundation for innovative, interdisciplinary approaches. The correlation between the surge in publications and the identification of shared pathological mechanisms suggests that research growth is driven by both scientific curiosity and clinical urgency, particularly as the population of aging HIV-positive individuals expands (5). The high rate of international co-authorship and collaboration between leading institutions highlights the importance of interdisciplinary and cross-border efforts in addressing complex questions related to HIV-AD comorbidity. The evolution from broad neurodegenerative studies to specific mechanistic and therapeutic investigations reflects advancements in technology and a deeper understanding of the biological interfaces between HIV and AD, indicating a field that is both maturing and specializing.

International collaboration appears to drive innovation and knowledge consolidation, particularly in high-income countries with well-funded research infrastructures. Collaborative research may enhance the quality and scope of studies, such as those exploring neuroinflammation and biomarkers in HIV-AD comorbidity (38). The concentration of research in high-income countries may limit the generalizability of findings to low- and middle-income settings. Strengthening collaboration with underrepresented regions could address gaps in understanding the sociocultural and environmental factors influencing HIV-AD interactions.

A notable decline in publication output after 2019, particularly in 2024 and 2025, coincides with the COVID-19 pandemic and potential shifts in research priorities. This decline may indicate resource reallocation toward pandemic-related research or a transition toward more specialized or underfunded niches within HIV-AD research. The COVID-19 pandemic likely diverted funding and attention away from HIV-AD research, though its long-term impact remains unclear (39). The decline could reflect a maturation of the field, with researchers focusing on deeper mechanistic questions rather than broad descriptive studies. Reduced publication output may highlight funding gaps, particularly for studies integrating advanced technologies like machine learning.

Seminal papers by Lozano et al. (17) and Naghavi et al. (18) have significantly influenced the field, with thousands of citations and sustained academic impact. These studies have shaped research trajectories by emphasizing the epidemiological and pathological overlaps between HIV and AD, guiding subsequent investigations into neuroinflammation and biomarkers. High-impact papers have laid the groundwork for understanding how HIV-associated neuroinflammation contributes to AD-like pathologies (40). Studies with citation bursts (21) have driven specific research directions, such as amyloid precursor protein mechanisms. The enduring relevance of these papers underscores the importance of addressing unanswered questions in HIV-AD pathogenesis.

Keywords like “neuroinflammation” and “microglia” emerged as central themes indicating a focus on inflammatory pathways in HIV-AD interactions (41). Neuroinflammation is a critical mediator of neuronal damage in both HIV and AD highlighting its potential as a therapeutic target (42). Shared inflammatory pathways suggest that interventions targeting neuroinflammation could benefit both conditions (43). Research on microglial activation and cytokine profiles may inform early diagnostic strategies (44). Understanding neuroinflammation could guide the development of combination therapies for HIV-positive individuals at risk of AD.

Keywords such as “beta-amyloid” and “tau protein” reflect sustained interest in protein aggregation mechanisms. These proteins are central to AD pathogenesis and may play roles in HIV-associated neurodegeneration particularly in aging populations (45). Amyloid-beta and tau pathology may exacerbate cognitive decline in HIV-positive individuals necessitating integrated diagnostic approaches (46). Targeting amyloid-beta and tau could offer dual benefits for HIV-AD comorbidity (47). The growing population of aging HIV patients requires urgent research into age-related neurodegeneration (48).

Recent trends highlight emerging themes like “machine learning” and “extracellular vesicles,” indicating a shift toward data-driven and translational research (49). These trends suggest that future research should prioritize mechanistic studies, biomarker validation, and longitudinal assessments of at-risk populations. Investigating the role of specific inflammatory mediators in HIV-AD interactions could yield novel therapeutic targets (50). Exploring how genetic factors influence vulnerability to neurodegeneration in HIV-positive individuals may inform personalized medicine approaches (51). Prioritizing longitudinal studies to assess AD risk in aging HIV cohorts and evaluate preventive strategies is essential for advancing clinical practice (52).

This bibliometric analysis reveals a dynamic and rapidly evolving research landscape at the intersection of HIV and AD, marked by a 14.18% annual growth in publications and significant international collaboration. Key research themes include neuroinflammation, protein aggregation, and blood–brain barrier disruption, reflecting shared pathological mechanisms between HAND and AD (53). For researchers, this analysis identifies critical gaps and future directions, such as investigating the role of neuroinflammation in HIV-AD interactions, exploring genetic susceptibility factors, and leveraging advanced technologies like machine learning for biomarker discovery. The identification of emerging research clusters and citation bursts provides insights into the field’s evolving priorities, guiding resource allocation and study design. For clinicians, the findings emphasize the need for integrated approaches to manage aging HIV-positive populations at risk of AD (54). Longitudinal assessments to monitor cognitive decline, early diagnostic strategies targeting shared biomarkers, and therapeutic interventions addressing neuroinflammation and protein aggregation are highlighted as essential steps (6). The analysis also underscores the importance of considering the blood–brain barrier in developing preventive and treatment strategies.

This analysis provides key insights into the intersection of HIV and AD but has methodological limitations. The study relied solely on the Web of Science Core Collection, which may introduce regional and funding biases (55). This could potentially affect the generalizability of the findings, especially for low- and middle-income countries (56). Excluding PubMed and Scopus might result in missing studies from biomedical journals and regional research outputs not indexed there (57). PubMed offers comprehensive biomedical coverage and free access, while Scopus excels in global research outputs (58), particularly non-English and regional journals (59). Omitting these databases could limit the comprehensiveness of the findings, especially in underrepresented regions or specific biomedical fields (60). Future research should include these databases to provide a more complete view. This would enhance methodological transparency and aid readers in interpreting the scope and generalizability of the findings. Such an approach would better capture the full spectrum of research, particularly from non-English-speaking regions and emerging research areas, ensuring a more balanced and inclusive representation of global efforts in this critical field (61). Overall, this study offers a roadmap for advancing both research and clinical practice. It fosters innovation through interdisciplinary collaboration and translates scientific discoveries into improved patient outcomes.

## 5 Conclusion

This study provides a concise bibliometric analysis of research trends at the intersection of HIV and AD, revealing a dynamic research landscape with a 14.18% annual growth in publications. High-income countries, particularly the United States, led in research output, with key themes including neuroinflammation, protein aggregation, and blood–brain barrier disruption. HIV promotes AD through neuroinflammation induced by microglial activation, leading to amyloid-beta and tau protein accumulation (1). HIV proteins like Tat disrupt synaptic function, while blood–brain barrier disruption allows immune cell infiltration, exacerbating neuroinflammation (1). Oxidative stress from HIV infection accelerates amyloid-beta aggregation and tau hyperphosphorylation (62). Emerging research highlights the role of extracellular vesicles in spreading pathology and the potential of advanced technologies like machine learning in biomarker discovery (63). Future research should focus on longitudinal studies to assess AD risk in aging HIV populations, investigate specific inflammatory mediators, and explore genetic factors (64). Addressing these gaps through interdisciplinary collaboration will help translate scientific insights into improved clinical outcomes for aging HIV-positive individuals at risk of AD.

## Data Availability

The raw data supporting the conclusions of this article will be made available by the authors, without undue reservation.
